# The Effect of General Conditions on the Quality of the Teeth

**Published:** 1916-12

**Authors:** W. R. Ackland

**Affiliations:** President of the Bath and Bristol Branch of the British Medical Association


### THE EFFECT OF GENERAL CONDITIONS ON THE QUALITY OF THE TEETH.

The Presidential Address, delivered on June 30th, 1915, at the opening of the Session of the Bath and Bristol Branch of the British Medical Association.

BY

MAJOR W. R. ACKLAND, R.A.M.C. (T.),

*President of the Bath and Bristol Branch of the British Medical Association.*

---

THE choice of a subject for my address is not easy Something of general interest on which I can say anything new is difficult to find. Still thirty years' experience tends to form opinions which may be worth something; but I confidently ask your indulgent criticism in consideration of the great preoccupation of my time by my duty at the Military Hospital.

Much has been said and written of the *general troubles to which dental disease may give rise.* But while the subject is not threadbare, I want to talk to you to-night on rather the converse, namely the *effect of general conditions on the quality of the teeth.*

As a preliminary, I should like to recall to your memory that we regard caries of the teeth as being immediately due to one or both of two factors.

First of all a poor quality of tooth, and secondly unfavourable surroundings in a mouth whose secretions are vitiated. The teaching of pathologists in my time was rather in favour of supposing that a tooth was born either good or bad, and that its quality never varied throughout life. The secretions of the mouth might possibly vary from time to time, but as far as one could make out the tooth was a passive agent at the mercy of those secretions.

Now I hope to induce you to take a point of view which is the result of many years of clinical observation, namely that *a tooth is not a passive agent, that is to say, it is capable of vital resistance which varies according to the health of the patient, very much as we find the vital resistance in any other tissue does.*

I was led early to take this view from the fact that Bödecker, a well-known histologist, had attempted to prove that not only the dentine but also the enamel is penetrated everywhere by a network of living plasm derived from, though far finer, than the dentinal fibrils. More recently Mummery has demonstrated what every patient long ago knew to exist, namely a fine extension of nerve elements throughout the dentine right up to the under surface of the enamel. We should *a priori* expect this to be so in the case of a living tooth in a living body, but further consideration of other facts leads me to the strong opinion that *a tooth varies in quality from time to time according to the condition of health.*

And the reasons for my opinions are :—

1. First of all it is in the experience of us all that our teeth vary in sensitiveness from time to time, and I venture to assume that we may regard this as being due to the varying of the proportion of lime salts.

2. The existence of the " translucent layer." This is an appearance seen under the microscope surrounding decay which is supposed to be due to the sealing of the dentinal tubes by lime salts to prevent the nerve pulp being attacked.

3. The deposition of secondary dentine in the pulp chamber as a protection to the pulp, and the consequent withdrawal of the latter to a distance from the advancing decay.

4. The recalcification of softening areas in teeth which have started to decay. This is sometimes seen after the renewed health produced by a sea voyage.

5. The absorption and translucency of the roots of teeth attacked by pyorrhea. This is no doubt the result of a lowered vitality of the periosteum and tooth pulp. I believe I have done right in putting this before you at the start, because I do not see on what other theory we can explain the effects of many conditions on the teeth. We have to begin very early in considering what these conditions may be.

*Hereditation.*—Let us start with *hereditation.* Caries of the teeth may result from inheritance in various ways : (*a*) there may be a heritage of teeth of poor quality, or (*b*) of crowded jaws and irregularity, tending to produce disease, or (*c*) of congenital diatheses such as syphilis, gout, rheumatism, which undoubtedly act in producing caries in two ways, one by vitiation of the secretions, but perhaps more markedly by the tendency to lower the vital resistance of a tooth as they do of the rest of the body.

*Maternal conditions.*—Next in order I note the effect produced on a child's teeth by the ailments of pregnancy.

These effects chiefly make themselves felt by the temporary or baby teeth.

*Bottle feeding.*—I have no time to trace the cause which makes bottle feeding so bad for children's teeth, but you know that this is a well-established fact.

*Childish ailments.*—It is unnecessary for me to go closely into the explanation of the bad effect of childish ailments on the permanent set of teeth. I simply point out to you that the permanent set is being calcified during the first eight or ten years of life, and we may assume therefore that any lowering of health at various times by the diseases of childhood will leave their marks on the second set.

*Mouth breathing.*—The effects of mouth breathing have been brought to your notice at various times. The reason why this is so deleterious to the teeth is probably also known to you. Through the rapid evaporation, the teeth are no longer flushed with saliva, but are coated with an acid mucus instead.

*Surroundings of childhood.*—Now I should like to say a few words to you about the surroundings of childhood and the effects they produce on the quality of teeth. I am reminded to lay special stress on these from the following experience. Some years ago, during the life of a rich patient, we gave regular dental attention to the tenants' children on a model estate near Bristol. We were struck at starting by the fact that their teeth, though showing the usual neglect of their class, nevertheless evidenced little signs of decay. They lived in hygienic surroundings, and their parents being a selected class of sober habits with good wages, these children had good food, good air, and plenty of sleep. The case of these children affords the strongest additional evidence to my mind in favour of the theory that I was trying to prove just now, that a tooth is a very much more vital organ than we have been led to think, and that surroundings which make for general good health give us a high vital resistance in such teeth. Of course, the other point proved is that during the time the teeth are being developed and calcified it is very important that the child should be in the best of health.

But I want to go into the effect of surroundings in detail. I need not attempt to prove to you that *bad air* may produce ill effects on the health, nor shall I trouble you to follow the effects of *bad food* on the growing child. The conditions which unfortunately prevail in the slums of our cities have a wretched effect on the health of the children, and of course we should expect their teeth to be *pari passu* affected.

*Sleep.*—But I am going to lay the greatest stress possible on the fact that these children from babyhood upwards never get enough of " tired nature's sweet restorer, balmy sleep." I do not think sufficient importance has ever been given to the *want of sleep* in considering the conditions affecting the health of children in our slums. I have been coming up from the Hospital very late occasionally, and have constantly seen youngsters and even babies about at all hours.

From the remarks I have made before you can see two reasons why the teeth of these children should suffer in quality. First of all during childhood, while the calcification of the second set is going on, a lowered vitality prevents these developing teeth from getting their proper share of formative material. In the second place, when attacked by decay their power of resistance is very poor.

I had better include here an observation on the effect of the *deficiency of lime salts in soft water districts.* We should naturally expect to find the children's teeth suffer, and this I found to be the case at Porlock, where the drinking water is derived from moorland streams.

So far I have been dealing with the conditions affecting the teeth of children. But there is not much to add in the case of "children of a larger growth," viz. adults. Undoubtedly conditions affecting the general health act on the teeth of adults in a similar way, first by diminishing resistance, and secondly by vitiating the saliva.

*Effect of age.*—It is to be noted that the period of greatest decay is up to eighteen to twenty years. There is lessening decay up to the age of thirty. During middle life to fifty decay is least, and slightly increases again after that, if there are any teeth left, that is to say.

*Occupation.*—A few observations on the effect of *occupation* may be useful. I have noticed that workers in an atmosphere of sugar, starch or flour have their teeth very much affected, especially in the case of "mouth breathers," in whom the tidal air tends to dry up the saliva and deposit particles of these acid-producing agents on the teeth.

*Pyorrhea Alveolaris.*—I suppose just now no paper on the diseases of the teeth would be complete without a reference to *pyorrhea.* If I had been addressing you on the subject of general diseases caused by teeth, you would certainly expect to hear a good deal about it.

And in the present case, though I definitely stated that I was going to talk to you on the converse, namely the effect of general conditions on the teeth, the views I hold in regard to pyorrhea make me think it well within the scope of my title. In the greater number of cases I think pyorrhea is rather the effect than the cause. For example, rheumatism may and does cause pyorrhea many times more often than the other way round. There is not the slightest doubt in my mind that in many cases of commencing pyorrhea which I have seen amongst the wounded at the Hospital it has been due practically entirely to rheumatism and exposure in the flooded trenches ; and when my medical colleagues have in these cases of rheumatism sent for me to treat the teeth as one method of treating rheumatism, I have wondered very much why they sought further explanation of the rheumatism than their knowledge of the surroundings of these poor fellows last winter.

Some of these cases of commencing pyorrhea were clearly traceable to malaria, others to syphilis, some to mercury. Here I want to put on record that in my opinion the prevalence of pyorrhea is over-stated. I constantly get cases of supposed pyorrhea sent to me in which it does not exist at all, and I see innumerable cases of simple gingivitis in which there is no pus, and which therefore I should be disinclined to call pyorrhea.

In almost any person you like to examine pressure at the gum edge will extrude a whitish matter which some people mistake for pus. It is not so easily produced immediately after cleaning; in many cases it is simply dissolving debris of food which is lodged in the loose edges of the gums, especially when the teeth are not cleaned properly. Finally, in regard to pyorrhea, I am going to take full advantage of my position to-night to state most emphatically that in my opinion it is a dangerous obsession with many medical men and dentists, which is causing the loss of thousands of valuable teeth every day. The public papers have inoculated the lay mind with it, so that our patients have got it on the brain if they have not got it in their mouths. Every possible disease is attributed to this fancied condition. Even when there is no general disease present, teeth are condemned lest disease should arise. I am not saying that pyorrhea does not exist, I am not saying that it does not cause general symptoms in many cases, but I do repeat most seriously that cases are labelled pyorrhea which are nothing of the kind, and that valuable teeth are being sacrificed every day.

Let me give you an instance of the kind. Some years ago a well-known lady telegraphed from London for an appointment, and when she came she asked me to examine her mouth, as if she were one of my regular patients coming to me periodically for that purpose. I did so, and found as I told her that she had recently been to her dentist, that perhaps as a "counsel of perfection" one tooth might be stopped, and that she would be better off for a small upper plate carrying four upper teeth, two bicuspids on either side. I should add that she had lost no other teeth. She then asked me to examine again more carefully, especially with a view to saying what teeth I should extract. I did so, and said, "There are none." She then said, "What about pyorrhea?" I replied, "You have not got it." There was a certain amount of recession of the gums associated with her age, which was fifty-eight, but there was no evidence whatever of pus or any congestive condition. She then proceeded to tell me her story. She had gone to her regular dentist for examination, and as he was away his partner examined her mouth, and started off by entreating her to have every tooth out. Of course I naturally asked if she had any symptoms of ill-health which induced him to take such an extreme view. "No," she said, "I am perfectly well, and the question of my health never arose." Well, to make a long story short, she kept her teeth, and I am able to tell you that after these many years she not only has them still, but her general health is as good as ever.

You will note that in speaking of the effect on teeth produced by congenital diatheses or the ailments of childhood I have made no reference to Hutchinson's teeth, gouty teeth, etc. As a matter of fact, we have found that it is misleading to associate definitely any form of ill-developed or, as we call it, hypoplasic tooth with a special disease. For instance, there is a good deal of congenital syphilis without Hutchinson's teeth, and there have been many ill-developed teeth called Hutchinson's teeth in cases where there was no congenital syphilis.

So far I have been dealing firstly with the general conditions which affect the development of teeth of good quality, and secondly with those conditions which maintain a good resistance. In other words, we have been considering the conditions affecting the defence to the exclusion of those affecting the attacking forces. In proceeding to discuss the latter I appear to be going beyond the scope of my title, for I am introducing some remarks on what is after all a consideration of local conditions. Yet as many of these are the result of the action of general conditions, I feel my address will be incomplete without them, but I will be as concise as possible.

What are the conditions of the mouth which constitute the attacking forces ?

*Briefly, they are the presence of acid-forming organisms in the mouth acting on fermentable carbo-hydrates.*

Before I discuss the possibility of preventing caries I should like to recapitulate a point or two. Firstly, that the development of good-quality teeth is dependent on :—

(*a*) A good inheritance.
(*b*) Good health in the mother.
(*c*) Hygienic surroundings in the child.
(*d*) Good, suitable food at regular times.
(*e*) Plenty of sleep.

Secondly, that the quality of the tooth once formed may vary with the health, especially during the years of growth.

Finally, I have to consider, as I said above, what promise there is of our being able to prevent decay altogether. Given a good inheritance and a good environment, a child ought to develop strong teeth with a good vital resistance.

Theoretically, according to Pickerill, it ought to be possible, having set up such good defence, to ward off the forces of attack, and the first thing to note is (*a*) the prevention of thumb-sucking, etc., and especially of comforters, inasmuch as these tend to restrict the development of the jaw. (*b*) Children should be encouraged to eat hard food on account of its beneficial effect on the periodontal membrane nd also upon jaw development. We may regard the saliva as protective against the occurrence of caries. Substances which provoke the flow of the saliva, such as acids and pleasant flavours with the food, are beneficial. (*c*) In order to prevent the retention of fermentable carbo-hydrates, starches and sugars should not on any account ever be eaten alone, but in all cases be combined with a substance having a distinctly acid taste, such as the natural organic acids found in fruit and vegetables. He further recommends an acid mouth-wash, which is entirely opposed to our preconceived ideas.

Pickerill sums up his observation on dietary by saying that all meals should contain a fair proportion of salivary excitants, and, more important still, should both commence and end with some article of diet having an acid reaction. He recommends that every meal should conclude with fresh fruit. He strongly condemns tea and the usual carbo-hydrates which accompany it.

Last thing at night he suggests a small portion of such fruits as orange, apple, pear or pineapple, apparently with the sole object of provoking a good flow of saliva.

Children should be encouraged to spend their pocket-money on fruit rather than on sweets, and on biscuits least of all. The final cleaning at night must of course be most carefully observed, and a word of caution is necessary that it should be absolutely the last thing. I have frequently found when questioning parents that they regard milk or biscuits taken in bed (*i.e.* after the cleaning) as not affecting the case.

